# Genome-Wide Association Studies and Genomic Selection in Pearl Millet: Advances and Prospects

**DOI:** 10.3389/fgene.2019.01389

**Published:** 2020-02-28

**Authors:** Rakesh K. Srivastava, Ram B. Singh, Vijaya Lakshmi Pujarula, Srikanth Bollam, Madhu Pusuluri, Tara Satyavathi Chellapilla, Rattan S. Yadav, Rajeev Gupta

**Affiliations:** ^1^International Crops Research Institute for the Semi-Arid Tropics (ICRISAT), Hyderabad, India; ^2^All India Coordinated Research Project on Pearl Millet (AICRP-PM), Indian Council of Agricultural Research (ICAR), Jodhpur, India; ^3^Institute of Biological, Environmental & Rural Sciences (IBERS), Aberystwyth University, Gogerddan, United Kingdom

**Keywords:** pearl millet, genetic resources, genomic resources, genomic selection, genome-wide association studies, molecular markers

## Abstract

Pearl millet is a climate-resilient, drought-tolerant crop capable of growing in marginal environments of arid and semi-arid regions globally. Pearl millet is a staple food for more than 90 million people living in poverty and can address the triple burden of malnutrition substantially. It remained a neglected crop until the turn of the 21st century, and much emphasis has been placed since then on the development of various genetic and genomic resources for whole-genome scan studies, such as the genome-wide association studies (GWAS) and genomic selection (GS). This was facilitated by the advent of sequencing-based genotyping, such as genotyping-by-sequencing (GBS), RAD-sequencing, and whole-genome re-sequencing (WGRS) in pearl millet. To carry out GWAS and GS, a world association mapping panel called the Pearl Millet inbred Germplasm Association Panel (PMiGAP) was developed at ICRISAT in partnership with Aberystwyth University. This panel consisted of germplasm lines, landraces, and breeding lines from 27 countries and was re-sequenced using the WGRS approach. It has a repository of circa 29 million genome-wide SNPs. PMiGAP has been used to map traits related to drought tolerance, grain Fe and Zn content, nitrogen use efficiency, components of endosperm starch, grain yield, etc. Genomic selection in pearl millet was jump-started recently by WGRS, RAD, and tGBS (tunable genotyping-by-sequencing) approaches for the PMiGAP and hybrid parental lines. Using multi-environment phenotyping of various training populations, initial attempts have been made to develop genomic selection models. This mini review discusses advances and prospects in GWAS and GS for pearl millet.

## Introduction

Pearl millet (*Pennisetum glaucum* (L) R. Br., syn. *Cenchrus americanus* (L.) Morrone) is an important C_4_ small-grained field crop of traditional smallholder farming systems that belongs to the grass family *Poaceae* and subfamily Panicoideae. An archaeological survey indicates that pearl millet was initially domesticated at the southern edge of the Sahara Desert in West Africa about 2500 BC (Manning et al., 2011). Pearl millet is a diploid (2*n* = 2*x* = 14), cross-pollinated warm-season crop with tremendous photosynthetic potential and high biomass production capacity. It is highly tillering, polymorphic, has a short life cycle, a large genome size (1.76 Gb), and an outbreeding nature (Bennett et al., 2000, Varshney et al., 2017). Climate-adaptive phenotypic, physiological, and reproductive attributes of pearl millet make this crop well-suited to grow in marginal conditions, such as poor soil fertility, limited soil water content, high salinity, extreme soil pH ranges, high soil Al^3+^ saturation, high temperatures, and scant rainfall. Pearl millet can thrive and produce a substantial amount of grain in drought-prone areas that receiving average annual precipitation <250 mm, whereas other cereal crops, such as maize, rice, sorghum, bread wheat, and barley, are likely to fail to give economic returns (Nambiar et al., 2011). Pearl millet is cultivated over ~27 million hectares in arid and semi-arid areas of Asia and Sub-Saharan Africa and is the primary food source for about 90 million resource-poor populations residing in marginal areas globally. Remarkably, the natural attribute of this crop to withstand ambient temperatures up to 42°C at the reproductive phase makes it suited for growth *via* irrigation in the extremely hot summers in north-western parts of India (Gupta et al., 2015).

Pearl millet has several nutritional properties compared to other staple cereal grains, and it is an excellent source of organic as well as inorganic nutrients and a cost-effective source of energy (Kumar et al., 2016). Pearl millet grains are rich in fibers (1.2 g/100 g), α-amylose, amino acids, proteins (8–19%), and low starch, mineral nutrients including phosphorus, magnesium, iron, and zinc. Owing to having such nutritional values, pearl millet ensures food and nutritional security for farmers living in poverty (Nambiar et al., 2011, Kanatti et al., 2014). Pearl millet is a rich source of several polyphenols, and other biologically important ingredients make it suited to play a role in reducing the rate of fat absorption, the lowering of glycemic indices, as well as in overcoming the risk of cardiac diseases, diabetes, and other medical problems. Overall, pearl millet has the capacity to combat micronutrient deficiency across developing countries (Rai et al., 2012) since it contributes 30–40% of inorganic nutrients and provides affordable staple food with an adequate level of iron and zinc in its cultivating areas (Rao et al., 2006).

An alternative approach to the QTL mapping is the genome-wide association study (GWAS) or association mapping (AM) approach (Gómez et al., 2011) based on the principle of a linkage disequilibrium (LD) to detect a substantial association between DNA marker and target trait (Gupta et al., 2005). Genetic linkage is found through extensive genotyping of a panel of germplasm or breeding populations showing contrasting phenotypes across variable environments. It has an immense power in identifying specific genes controlling the expression of the desired traits (Kraakman et al., 2004). The potential advantage of association mapping is the likelihood of a superior resolution mapping utilizing mass recombination events from numerous meiotic events throughout the germplasm evolutionary history. It has the power to evaluate and characterize several alleles concurrently in diploid (Zhao et al., 2007) as well as in polyploid crops (Breseghello and Sorrells, 2006). Association mapping offers many benefits over linkage mapping since it provides better mapping resolution due to historical mutations and recombinations in genetic lineages, which leads to the identification of markers in the vicinity of governing genes (Liu et al., 2016). Genetic polymorphisms having strong linkage with a genomic locus leading to phenotypic differences is expected to be substantially associated with a target trait across the panel of germplasm.

The analysis of QTL effects for minor QTLs using linkage mapping and genome-wide association mapping is often biased. Therefore, scientific groups have for years been trying to solve the issue of how to tackle these complex traits and outcomes in terms of genomic selection (GS). Genomic selection is a breeding approach exploiting high-density DNA markers distributed across the genome to facilitate the rapid selection of the best candidates and offers opportunities to enhance genetic gains (Meuwissen et al., 2001). GS uses different prediction models by combining the genotyping and phenotyping datasets of the training population (TP), which is subsequently used to determine genomic-estimated breeding values (GEBVs) for every genotype of breeding population (BP) from their genotyping scores. These GEBVs permit breeders to envisage superior genotypes that would be suitable either as a parent in hybridization or for next-generation advancement of the breeding program. The basic principle is that the information derived from several markers widely distributed over the genome, having the potential to reveal genetic variations in the genome, can evaluate breeding values without prior information of where the selected genes are located (Crossa et al., 2017).

In this paper, we review the advances made in the development of genetic and genomic resources for their use in genome-wide association studies (GWAS) and genomic selection (GS) in pearl millet.

## Development of Genetic Resources

Genetic resources are the fundamental materials that play a pivotal role in plant genomic and phenomic studies to boost major scientific discoveries in advanced agriculture systems. Fortunately, genetic recourses have been collected and preserved by many national and international gene banks around the world. Pearl millet accessions have been collected and conserved by 97 gene banks (66,682 accessions) globally, in which ICRISAT has the largest collection (~21,594 pearl millet accessions from 51countries) (Singh and Upadhyaya, 2016). More importantly, core and mini core collections have been developed at ICRISAT and serve as essential resources for allele mining studies for the identification of agronomic studies, and they are also used for the development of tolerant lines for both abiotic and biotic stresses. Likewise, one more genotype-based reference set has been developed, and it comprises 300 pearl millet accessions (Upadhyaya et al., 2011). At ICRISAT, most of the accessions were evaluated for several agronomical traits, and these show the extent of genetic diversity and phenotypic variance for most of the qualitative and quantitative traits (Singh and Upadhyaya 2016). It is evident that vast genetic variability is the determining factor for the identification of promising germplasm for the desired trait (Upadhyaya et al., 2007). In addition to ICRISAT, major germplasm are preserved at the Institute of Research for Development (IRD, France), in which 3,968 accessions are maintained from 16 countries, and 3,821 accessions of cultivated *P. glaucum* and related species maintained at the Canadian Genetic Resources (Saskatoon, Canada). Additionally, there are 1,283 active collections of pearl millet accessions collected and preserved at the US Germplasm Resource Information Network (GRIN) (Yadav et al., 2007). For conducting AM studies, diverse genetic resources are the essential inputs, and pearl millet genetic resources are found to have enormous genetic diversity. For this reason, performing AM studies for desired traits in pearl millet crops is imperative and will provide immense genomic resources for future studies. Over the last five years, significant work has been carried out on pearl millet related AM studies, and this gives information about genetic diversity and linkage disequilibrium (LD). To get over this problem, ICRISAT, in association with AU, developed a world association mapping panel called the Pearl Millet inbred Germplasm Association Panel (PMiGAP). This panel comprises 346 lines consisting of germplasm lines, landraces, and breeding lines representing global pearl millet diversity. These lines were generated by repeated rounds of selfing (S_0_ through S_11_) from 1,000 accessions representing diverse cultivars, landraces, and mapping population parents of 27 countries. Thus, PMiGAP may be considered an excellent genetic resource for GWAS studies into pearl millet crop. By the year 2015, out of 346 PMiGAP lines, Sehgal used 250 lines for AM studies and evaluated these for drought-related traits under field conditions. Similarly, during another study on AM, in which 500 pearl millet lines included 252 global accessions and 248 Senegalese landraces, they found extant genetic diversity between global and Senegalese accessions (Hu et al., 2015). In addition to the above studies, several RIL (recombinant inbred line) populations were also developed for biotic and abiotic stresses, quality, as well as yield and yield-related traits. Rajaram et al. (2013) constructed pearl millet consensus maps by using four RIL populations (ICMB 841-P3 × 863B-P2 (RIP A), H 77/833-2 × PRLT 2/89-33 (RIP B), 81B-P6 × ICMP 451-P8 (RIP C), and PT 732B-P2 × P1449-2-P1 (RIP D). In other studies, iron- and zinc-related QTLs were identified in ICMB 841-P3 × 863B-P2 (144 progenies) and ICMS 8511-S1-17-2-1-1-B-P03 × AIMP 92901-S1-183-2-2-B-08 (317 progenies) RIL populations, respectively (Kumar et al., 2016; Kumar et al., 2018). In a recent study, Chelpuri et al. (2019) identified QTLs with resistance to major pathotype isolates of the downy mildew pathogen in the pearl millet RIL population, ICMB 89111-P6 × ICMB 90111-P6 (187 progenies). Therefore, there is a good opportunity for pearl millet researchers who can access these useful genetic resources to meet their research needs.

## Development of Genomic Resources and Trait Mapping

Molecular or DNA-based markers, genetic linkage maps, and genomic sequence data are important genomic resources to perform a genetic evaluation and marker-assisted breeding in any plant species. Over the last decade, several types of molecular markers, genomic tools, and genetic linkage maps have been developed and deployed in millets (Serba and Yadav, 2016). Several DNA-based molecular markers, including restriction fragment length polymorphism [RFLP; (Liu et al., 1994)], amplified fragment length polymorphism [AFLP; (Devos et al., 1995)], random amplified polymorphic DNA (RAPD), expressed sequence tags-derived simple sequence repeats [EST-SSRs; (Senthilvel et al., 2008; Rajaram et al., 2013)] markers, sequence-tagged sites [STSs; (Allouis et al., 2001)], genomic simple sequence repeat [gSSRs; (Qi et al., 2004)], DArT array Technology [DArTs; (Senthilvel et al., 2010; Supriya et al., 2011)], conserved intron specific primers [CISP; (Sehgal et al., 2012)], single-stranded conformation polymorphism-SNP [SSCP-SNP; (Bertin et al., 2005)], and single nucleotide polymorphisms [SNPs; (Sehgal et al., 2012)] have been developed and exploited in genetic diversity, QTLs/genes identification, and marker-aided breeding for faster pearl millet breeding (**Table 1**). Molecular markers facilitate in analyzing genetic variations existed within the germplasm collections for precise selection of breeding parents in crossing programs, estimating population structure, and identification of QTLs for stress tolerance. Pearl millet has a wide range of DNA polymorphisms even in elite inbred parental lines of popular hybrids (Vadez et al., 2012).

**Table 1:** Details of mapped traits and genomic resources developed in pearl millet, related to grain quality, yield, fodder, biomass, and biotic and abiotic stresses.

Sl. No.	Mapped traits	Reference
1.	Reported large-effect Fe and Zn content QTLs using DArT and SSRs markers to construct a genetic linkage map with 317 RIL population developed from ICMS 8511-S1-17-2-1-1-B-P03 × AIMP 92901-S1-183-2-2-B-08 cross.	Kumar et al., 2018
2.	Pearl millet genome sequencing data was used to establish marker trait associations for genomic selection, to define heterotic pools, and to predict hybrid performance.	Varshney et al., 2017
4.	A set of 305 loci were used to construct a linkage map to map two QTLs for grain Fe content on LG3 and LG5 and two QTLs for grain Zn content on LG3 and LG7 using replicated samples of 106 pearl millet RILs (F6) derived from ICMB 841-P3 × 863B-P2 cross.	Kumar et al., 2016
5.	Identified 83,875 SNPs within 500 pearl millet accessions, consisting of 252 accessions and 248 Senegalese landraces, with genotyping by sequencing (GBS) of PstI-MspI reduced representation libraries.	Hu et al., 2015
6.	Thirty-seven SSRs and CSIP markers have been developed, spanning 7 LGs evaluated in irrigated and drought stress conditions,22 SNPs, and 3 InDels for abiotic stresses	Sehgal et al., 2015
7.	ISSR-based SCAR marker has been devised for downy mildew (DM) resistance in pearl millet and associated to DM resistance LG with genetic linkage distance of 0.72 cM	Jogaiah et al., 2014
8.	Seventy-five SNPs and CISP were developed from EST sequences using parents of two mapping populations for 18 genes	Sehgal et al., 2012
9.	Hundreds of polymorphic EST-derived SSRs were developed and deployed in mapping of RIL populations in pearl millet	Rajaram et al., 2010; Rajaram et al., 2013
10.	About 300 DArT markers have been used for the polymorphic in different pearl millet RIL populations	Senthilvel et al., 2010
11.	Cross-transferability of the 31-finger millet EST-SSRs were evaluated and found to be polymorphic in pearl millet	Arya et al., 2009
12.	Four EST-derived SSRs and 9 CISPs were used in linkage mapping using biparental mapping populations of pearl millet	Yadav et al., 2008
	A panel of 21 functionally informative EST-based SSRs and 6 gSSRs were developed in pearl millet	Senthilvel et al., 2008
13.	Nineteen EST-SSRs, among them 11 amplified and 4 were an appeared polymorphism on agarose gels	Yadav et al., 2007
14.	Sixteen EST-based polymorphic SSR markers	Mariac et al., 2006
14.	SSCP-SNP primes were developed through a comparison of rice and pearl millet EST collections	Bertin et al., 2005
15.	Thirty-six genomic SSRs were developed from genomic clones	Qi et al., 2004
16.	Genetic maps developed in four different crosses were integrated to generate a consensus map of 353 RFLP and 65 SSR markers.	Qi et al., 2004
17.	Eighteen potential SSR markers were developed from genomic sequences in pearl millet	Budak et al., 2003; Allouis et al., 2001
18.	RFLP probes were used to assess genetic diversity within and between 504 landraces of core collection using a subset comprising 10 accessions of Indian origin	Bhattacharjee et al., 2002

Initially, RFLP-derived DNA markers were devised and used to map about 180 loci ranged approximately 350 cM under seven linkage groups in pearl millet (Liu et al., 1992; Liu et al., 1994). Later, these markers were exploited in QTL mapping for downy mildew resistance in pearl millet (Jones et al., 1995). A subset of 21 polymorphic EST-SSRs and 6 genomic SSR markers were developed using sequence information from 3,520 expressed sequence tags (ESTs) and used in genome mapping of different pearl millet mapping populations (Senthilvel et al., 2008). Subsequently, these potentially developed EST-SSRs were deployed in marker-aided breeding for yield and drought stress resistance in pearl millet at the International Crops Research Institute for the Semi-Arid Tropics (ICRISAT). The development of a panel of 277 polymorphic DArT markers was reported from 6,900 DNA array-dart technology (DArT) clones using a PstI/BanII complexity reduction in a pearl millet RIL population (Senthilvel et al., 2010). Separately, 574 potential DArT markers were detected from 7,000 DArT clones obtained from 95 diverse genotypes using a PstI/BanII complexity reduction in genetically diverse inbred lines of pearl millet (Supriya et al., 2011). The mapping of 208 DArT markers along with 305 SSRs detected seven linkage groups covering 1,749 cM with an average intermarker distance of 5.73 cM and two co-localized QTLs for iron and zinc content on LG 3 were identified in pearl millet (Kumar et al., 2016). Using DArT markers, comparative mapping and genome organization analysis may easily be performed, and the price of marker-aided backcrossing (MABC) is also cheap relative to other markers systems.

Pearl millet EST resources were used to develop quality SNPs and CISP markers, and they were deployed to identify candidate genes related to a major QTL for drought tolerance using diverse (H 77/833-2, PRLT 2/89-33, ICMR 01029, and ICMR 01004) genotypes that represented mapping populations parents (Sehgal et al. (2012). Later, 83,875 SNP markers were identified using genotyping-by-sequencing (GBS) of *Pst*I-*Msp*I reduced representation libraries in pearl millet lines, represented by 252 world germplasm accessions and 248 landraces from Senegal, which revealed wide genetic variability in comparison to other germplasm collection in Africa and Asia (Hu et al., 2015). Moreover, ISSR-based sequence characterized amplified region (SCAR) markers were devised to examine genetic variations between two (ICMR 01007 and ICMR 01004) genotypes of pearl millet and a contrast mapping population for downy mildew resistance. A polymorphic locus (1.4 kb size) was found in the ICMR 01004 genotype, and further PCR amplification of these polymorphic loci was produced to be closely associated with downy mildew resistant LG with a genetic distance of 0.72 cM. An identified SCAR marker was eventually validated using diverse pearl millet genotypes belonging to Asia and Africa, and the outcomes demonstrate that the marker was linked to downy mildew disease-resistant genotypes only (Jogaiah et al., 2014). The development of a linkage map was reported to integrate 256 DArT markers and 70 SSR markers and used to identify QTLs on LG1 with LOD score of 27 for rust resistance in 168 F_7_ pearl millet RILs derived from cross 81B-P6 × ICMP 451-P8 (Ambawat et al., 2016). Using a total of 106 pearl millet RILs (F6) derived from ICMB 841-P3 × 863B-P2 cross and 305 (96 SSRs and 208 DArT) markers, a linkage map was generated to map QTLs for grain iron and zinc content (Kumar et al., 2016). Recently, Kumar et al. (2018) reported a large-effect Fe and Zn content quantitative trait loci (QTLs) linked with DArT and SSR markers to construct a genetic linkage map using 317 RIL population derived from the (ICMS 8511-S1-17-2-1-1-B-P03 × AIMP 92901-S1-183-2-2-B-08) cross (**Table 1**).

## Case Studies for GWAS in Pearl Millet

The advent of the recently decoded pearl millet genome has opened prodigious possibilities to discern several QTLs and the functions of its associated candidate genes governing diverse traits (Varshney et al., 2017). The genome size of pearl millet ~1.79 Gb, representing 38,579 genes, 88,256 SSRs, and 4,50,000 SNPs, will certainly be a valuable resource for constructing precision genetic maps (Varshney et al., 2017). Genetic mapping can be constructed in two different ways; one way is through QTL-mapping/interval mapping (IM) and the other is by using the association mapping (AM)/LD-mapping approach. The major difference in these two mapping strategies is based on the presumed idea over recombination events causative for the phenotypic variations (Myles et al., 2009). In general, QTL-mapping/IM can be done by developing various mapping populations *viz*., F_2_, and recombinant inbred line (RIL), near-isogenic line (NIL), back cross (BC), and doubled haploid (DH)-derived populations in which one can assume a clear cut degree of relatedness for the recombination events between the two contrasting parents for the trait of interest (Abdurakhmonov and Abdukarimov, 2008). Genetic mapping in this type of controlled population size results in the limited attainability of meiotic events and the products in the form of QTLs will be localized with lower resolution (10 to 20 cM intervals), and it is also an expensive approach to maintain a large number of populations (Jannink and Walsh, 2002; Flint-Garcia et al., 2003; Holland, 2007).

On the other hand, in AM there is no requirement for developing hybridization-based mapping populations; rather, it needs diverse germplasm accessions, including collections of different land-races, varieties, and a breeding material termed as a ‘panel’ where relatedness for the recombination events are not under control because of numerous meiotic recombinations across the diverse germplasm (Verdeprado et al., 2018). The principle of AM relies on the linkage disequilibrium (LD), a non-random association between two genes/markers/QTLs at different loci; however, a non-random association between these components in the same loci results in increased linkage disequilibrium levels (Flint-Garcia et al., 2003; Álvarez et al., 2014). Taking the advantage of multiple historic recombination events within the diverse accessions since their domestication, the AM approach can be best suited for the identification of genes or QTLs with high resolution (100–1000 Kb), and these are tightly linked to a broad range of phenotypic traits (Mackay et al., 2009). The potential of identifying promising QTLs, and also in detecting causal polymorphisms at the gene level, has made association mapping a powerful approach to develop marker-trait associations (MTAs) with great precision (Meuwissen and Goddard, 2000; Palaisa et al., 2003).

**Figure 1:**
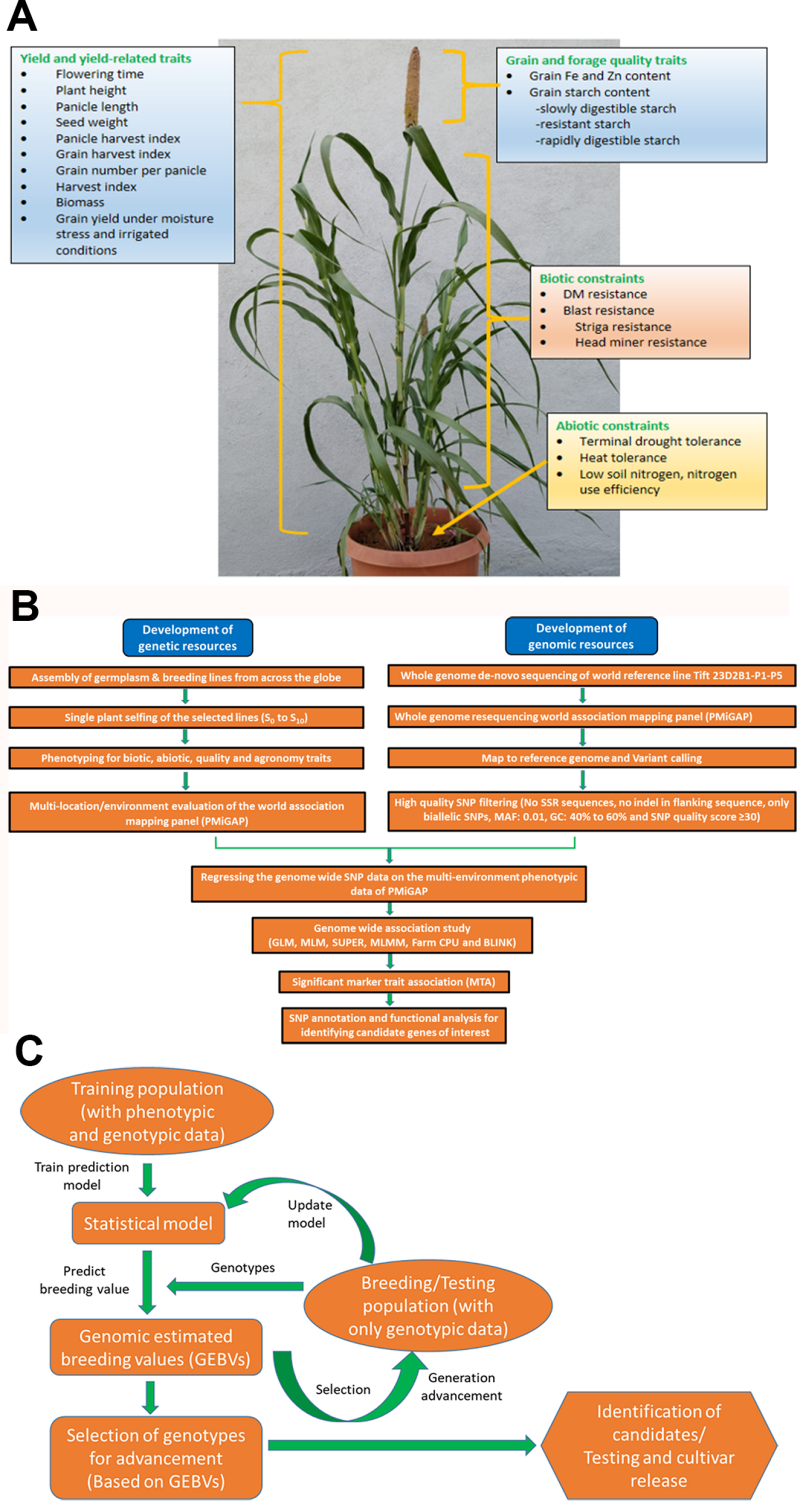
**(A)** Depiction of traits for which genome-wide association studies (GWAS) and genomic selection (GS) is being attempted at ICRISAT, Patancheru. **(B)** Workflow for genome-wide association studies (GWAS) pipeline. **(C)** Workflow for genomic selection pipeline.

However, due to the high level of heterogeneity and heterozygosity in most of the germplasm accessions of pearl millet, very few association mapping strategies were delivered (Kannan et al., 2014); herein they are discussed and these detailed approaches may expand the scope of AM studies of pearl millet in future. A generalized workflow for the pearl millet genome-wide association studies (GWAS) pipeline is presented in **Figures 1A, B**. Pearl millet crop adaptation to various agro-climatic conditions is an important subject of study to explore the underlying genetics associated with this important nutri-cereal. Association studies made by Saïdou et al. (2009) on this aspect reveals the genetic factors responsible for the variations in flowering time at the phytochrome C (PHYC) (866 bp) locus, which is one of the key trait involved in crop adaptation. A total of 90 inbred and 598 pearl millet varieties from India, East, and West Africa were used for generating phenotypic data; followed by genotyping with 27 SSR and 6 AFLP markers. An LMM (linear mixed model) was used to identify a significant association between the phenotypic trait and genetic variations. With an aim to identify the best candidate gene loci associated with the flowering time, Saïdou et al. (2014) further explored an extra 100bp region surrounding the PHYC gene and performed an association study, MCMC method (Markov chain Monte Carlo method), to identify the tightly linked markers (75 SNPs and INDELS) surrounding the PHYC (6 Kb) genomic region and also to show the extent of LD to confer *PHYC* gene as the best candidate gene. By integrating the genome scan approach with association mapping, Mariac et al. (2011) identified the *PgMADS11* gene, a MADS-box gene family member which plays a key role during somatic and reproductive phase development respective of different climatic conditions. Phenotyping data for the targeted traits from the 90 inbred lines *viz*., flowering time (FT), stem diameter (SD), plant height (PH), spikelet length (SpL), and spikelet density (SpD) are used for the association analysis; and the significant identified association of *PgMADS11* alleles with a varied flowering time that deciphers the role of *PgMADS11* in the plant adaptation process towards climatic change. Association studies of the selective SSR markers with the flowering time, plant height, panicle length, stover and grain yield were deciphered by Kannan et al. (2014).

A set of 250 full-sib progenies and 34 SSR markers were used for GWAS analysis, and results revealed the strong association of the *Xpsmp*2248_162 marker allele at linkage group 6 (LG6) with earlier flowering time and reduced plant height. Marker allele, *Xpsmp*2224_157 on LG7 was strongly associated with the plant height. For panicle length, *Xpsmp*2077_136, *Xpsmp*2233_260, and *Xpsmp*2224_157 were strongly associated with LG2, LG5, and LG7, respectively, whereas the *Xpsmp*2237_230 marker allele showed strong positive association on LG7 with grain yield. For stover dry matter yield, the *Xicmp*3058 193 marker allele showed strong positive correlation on LG6. There is a pressing need for information on genes associated with low phosphorus tolerance, especially in the regions of West Africa. Gemenet et al. (2015) made the first-ever reported association analysis of the available 285 DArT markers with the phenotypic data generated from 151 PMiGAP lines from West Africa across six environs under high and low P conditions. Results showed that the *PgPb11603* DArT marker showed stable association with the flowering time, and the *PgPb12954* marker showed a significant association with the grain yield.

Association studies reveal that *Xibmsp11/AP6.1*, an SNP marker on an acetyl CoA carboxylase gene, is strongly associated with GY, GHI (grain harvest index), and PY (panicle yield) under both treatments; whereas InDel markers *viz*., *Xibmcp09/AP10.1* & *Xibmcp09/AP10.2* of a chlorophyll a/b binding protein gene are associated with GY and stay-green traits. Using association mapping, key alleles for grain iron and zinc were demonstrated by Anuradha et al. (2017). Developing MTAs (Marker Trait Associations) between 250 SSR and 17 genic markers with grain iron and zinc content for 130 diversified lines across different environs revealed that the Xicmp3092 marker had a strong association with grain iron content on LG 7, and markers Xpsmp2086 & Xpsmp2213 and Xipes0224 showed association with grain zinc content on LG 4 and LG 6, respectively; conserved association for grain iron and zinc, however, was exhibited by Xipes0180, Xpsmp2261, and Xipes0096 on LG 3, LG 5, and LG 7, respectively. Another association study by Varshney et al. (2017) delivered key findings while establishing MTAs. Using whole-genome SNP data, a total of 3,117,056 SNPs were selected for GWAS analysis, and the phenotypic data for 20 agro-morphological traits was generated from 288 TCH (testcross hybrids) under two-stage (early & late) drought stress conditions along with respective controls. A significant association of the markers with the desired trait GNP (grain number per panicle) was exhibited on pseudomolecules Pg1 and Pg5. Genetic and genomic sequence information is now readily available for pearl millet. As AM will purvey a high-resolution power with the species exhibiting genotypic diversity across the germplasm (Álvarez et al., 2014), expanding AM studies in pearl millet will be increasingly fruitful for further crop improvement programs.

## Case Studies for Genomic Selection (GS) in Pearl Millet

Genomic (or genome-wide) selection (GS) is a promising strategy that has huge potential to explore and increase the genetic gain per selection in a breeding scheme per unit timeline and, thus, speed and efficacy in breeding programs (Spindel et al., 2015). GS has proven to be an economical and viable alternative to marker-assisted selection (MAS) and phenotypic selection (PS) for quantitative traits and accelerated crop improvement programs in cereals and several other crops (Heffner et al., 2009; Zhong et al., 2009; Crossa et al., 2010; Ornella et al., 2012; Poland et al., 2012; Spindel et al., 2015; Muleta et al., 2019). By developing efficient training population (having both genotypic and phenotypic data) designs, it predicts the genomic estimated breeding values (GEBV) of the testing population (having only genotypic data) by utilizing genome-wide high throughput DNA markers that are in linkage disequilibrium (LD) with QTL, and predicted GEBVs are used for selection (Meuwissen et al., 2001). One of the key advantages of GS is that decisions on selections can be taken during the off-season, leading to improvements in genetic gain on an annual basis (Heffner et al., 2009). Advancement and application of GS in pearl millet breeding programs facilitate precise prediction of hybrid performance along with ideal resource allocation. In ICRISAT, efforts are being made to exploit the available whole-genome resequencing (WGRS) data of PMiGAP lines along with phenotyping data for different traits for GWAS and GS. Building on the various target traits using GWAS (**Figures 1A, B**), various whole-genome prediction/genomic selection models are being developed and optimized in pearl millet. A generalized workflow for the pearl millet genomic selection pipeline is presented in **Figure 1C**.

Varshney and his group (Varshney et al., 2017) applied WGRS data for genomic selection by ridge regression best linear unbiased prediction (RR-BLUP) to predict grain yield for test crosses in four scenarios viz., the performance of grain yield in control, early stress, late stress, and across environments and observed high prediction accuracies for the performance of across environments. It was also reported that by using GS strategy (additive and dominance effects) the hybrid performance was also predicted by analyzing grain yield data with 302,110 SNPs, and 170 promising hybrid combinations were found, of which 11 hybrid combinations were already utilized for hybrid production with better performance and the remaining 159 hybrid combinations could be potential candidates for developing high yielding hybrids. A hierarchical clustering analysis of possible single cross combinations (167910) revealed two sets of lines with a higher hybrid performance by 8% by crossing each other. These hybrids could be a potential nucleus for establishing high-yielding heterotic gene pools for developing pearl millet hybrids with higher yield potential (Varshney et al., 2017). In a recent study, Liang et al. (2018) assessed two potential genotyping strategies viz., RAD-seq and tGBS, to characterize a set of ICRISAT-developed inbred pearl millet lines and evaluated the utility of genomic selection/prediction. By utilizing the projected hybrids from both (RADseq and tGBS) techniques and four genomic prediction schemes in pearl millet and assessed for each phenotype, 20 random rounds of five-fold cross-validation were performed for a tested SNP set. It was reported that, by utilizing hybrid data, the genomic prediction scheme (RR-BLUP) generated median prediction ranges (in parentheses) for different traits viz., 1,000 grain weight (0.73–0.74); days to flowering (0.87–0.89); grain yield (0.48–0.51); and plant height (0.72–0.73), respectively. Other traits with less/no heterosis, only hybrid, and hybrid/inbred schemes were also performed equivalently. It was also reported that hybrid GEBVs can be moderately improved by incorporating inbred phenotypic data sets, once inbred, and hybrid trait values relative to the mean trait values of that population. It was also well demonstrated that guileless integration of historical inbred phenotypic data into hybrid breeding programs could reduce the prediction accuracy of traits exhibiting heterosis. However, controlling the heterosis effects within the inbred genotype and trait data could improve the accuracy of GEBVs for hybrids, which, in turn, strengthens pearl millet hybrid breeding programs.

## Challenges in Using GWAS and GS for Pearl Millet

Being a poor man’s crop, pearl millet has attracted relatively less attention from various governments and policymakers in terms of support for the development of upstream science. This is particularly noted in areas such as GWAS and GS. The funding issues for carrying out this basic work in genomics has always remained an issue in pearl millet.

On the crop side, the high outcrossing rates, heterozygous nature, presence of inbreeding depression, and residual heterozygosity pose bottlenecks in inbred line development programs for the development of association mapping panels and for parental line/cultivar development were used in the training sets for GS. The presence of rapid linkage disequilibrium decay (LDD) warrants a relatively high number of markers for carrying out GWAS and GS. High rates of segregation distortions in specific populations may also pose serious challenges in GWAS and for getting high prediction accuracies for robust GS model development. Single-cross hybrids occupy a major market share in India, while top-cross and three-way hybrids are important for Africa. The development of GS models for hybrid parental lines resulting in heterotic combinations is quite challenging. These warrant precise estimation of the general combining ability (GCA) and specific combining ability (SCA) for specific agro-ecologies and their precise genotype-by-environment (G × E) interactions.

## Conclusions and Way Forward

Pearl millet is a nutritious, climate change ready crop capable of yielding economic return in marginal conditions where other cereals may fail. In recent years, pearl millet has seen an enormous increase in terms of various genetic and genomic tools at the disposal of pearl millet workers worldwide. Whole-genome sequencing of the pearl millet genome and resequencing efforts resulting in the generation of millions of genome-wide SNPs have facilitated efforts to map various yield and yield-related, key biotic and abiotic stress tolerance, and nutritionally important traits globally. These genomic resources have also facilitated taking up of the whole-genome prediction model development and validation efforts. There is a need to further validate the loci linked to various traits of interest and move from “loci” to “genes.” There is an enormous opportunity to apply these learnings in the development of robust whole-genome prediction models with special emphasis on combining ability and heterotic gene pool studies for the development of heterotic hybrids.

## Author Contributions

RKS and RG planned and coordinated this study. RKS, RG, RY, TC, RBS, SB, MP, and VP contributed to this work and drafted the manuscript. RKS and RG edited the manuscript for publication.

## Conflict of Interest

The authors declare that the research was conducted in the absence of any commercial or financial relationships that could be construed as a potential conflict of interest.
